# Intracranial hypertension and empty Sella from adrenal adenoma and excessive and prolonged steroid usage: a case report

**DOI:** 10.1186/s12902-021-00931-2

**Published:** 2022-01-15

**Authors:** Naiqian Zhao, Weixia Yang, Xiaoyan Li, Li Wang, Ying Feng

**Affiliations:** 1 Department of Gerontology, Second Hospital of Shanxi Medical University, Wuyi Road 382#, Taiyuan, 030001 China; 2 Department of Infectious Diseases, Jinzhong First Hospital of Shanxi Medical University, Huitong South Road 689#, Jinzhong, 030699 China

**Keywords:** Adrenal adenoma, Exogenous hypercortisolism, Intracranial hypertension, Empty Sella, Papilloedema

## Abstract

**Background:**

There is only one documented case of intracranial hypertension (IH) and empty sella from cortisol-producing adrenal adenoma so far. And IH and empty sella caused by long-term exogenous hypercortisolism has never been reported before. The purpose of this case report is to alert clinicians to glucocorticoid-induced IH.

**Case presentation:**

We present retrospectively a 50-year-old woman with cortisol-secreting adrenal adenoma, who progressed to intractable intracranial hypertension and a markedly expanded empty sella due to improper treatment. In 2011, the patient presented with hypertension, lack of cortisol circadian rhythm, low ACTH, a left adrenal adenoma and a partial empty sella, but did not receive low-dose dexamethasone suppression test (LDDST) and 24-h urinary cortisol. In 2014, she exhibited truncal obesity, raised cortisol, LDDST non-suppression, high urinary free cortisol and low ACTH, proving her cortisol-producing adrenal adenoma. She was simultaneously diagnosed with unexplained IH because of papilledema and elevated intracranial pressure, and her partial empty sella changed to a complete empty sella. In 2015, she underwent adrenal adenoma resection. From 2015 to 2018, she kept taking dexamethasone at least 2 mg daily without her doctors’ consent. During this period, she developed transient cerebrospinal fluid rhinorrhea, and her empty sella further worsened. After switching to low dose hydrocortisone, her papilledema disappeared completely, but optic atrophy has become irreversible.

**Conclusions:**

The patient seems to be just an extreme case, but it may reveal and illustrate a general phenomenon: Both cortisol-producing adrenal adenoma and long-term exogenous hypercortisolism could cause varying degrees of elevated intracranial pressure and empty sella. Clinicians should remain vigilant for this phenomenon in patients with cortisol-producing adrenal adenoma or excessive and prolonged steroid usage and give them corresponding examinations to identify this complication.

## Background

Idiopathic intracranial hypertension (IIH) is a clinical syndrome characterized by increased intracranial pressure of unclear etiology. Its most common pathological changes are papilledema and empty sella. If untreated or improperly treated, papilledema can lead to progressive and irreversible vision loss as a result of optic atrophy, and empty sella can cause hypothalamic-pituitary dysfunction and resultant hormonal alterations [1, 2]. Although the underlying cause of IIH is still unknown, many studies indicate that Cushing disease, chronic steroid administration and acute withdrawal of chronic steroid use are among the largely reported possible causal and associated risk factors [3]. In 1980, Britton et al. reported that there is an association between IIH and cortisol-secreting adrenal adenoma [4]. Since then, however, no other cases associated with cortisol-secreting adrenal adenoma have been reported. It is unknown whether IIH with cortisol-secreting adrenal adenoma has not been given due attention by the academia or it’s just an individual and accidental phenomenon.

## Case presentation

**Fig. 1:**
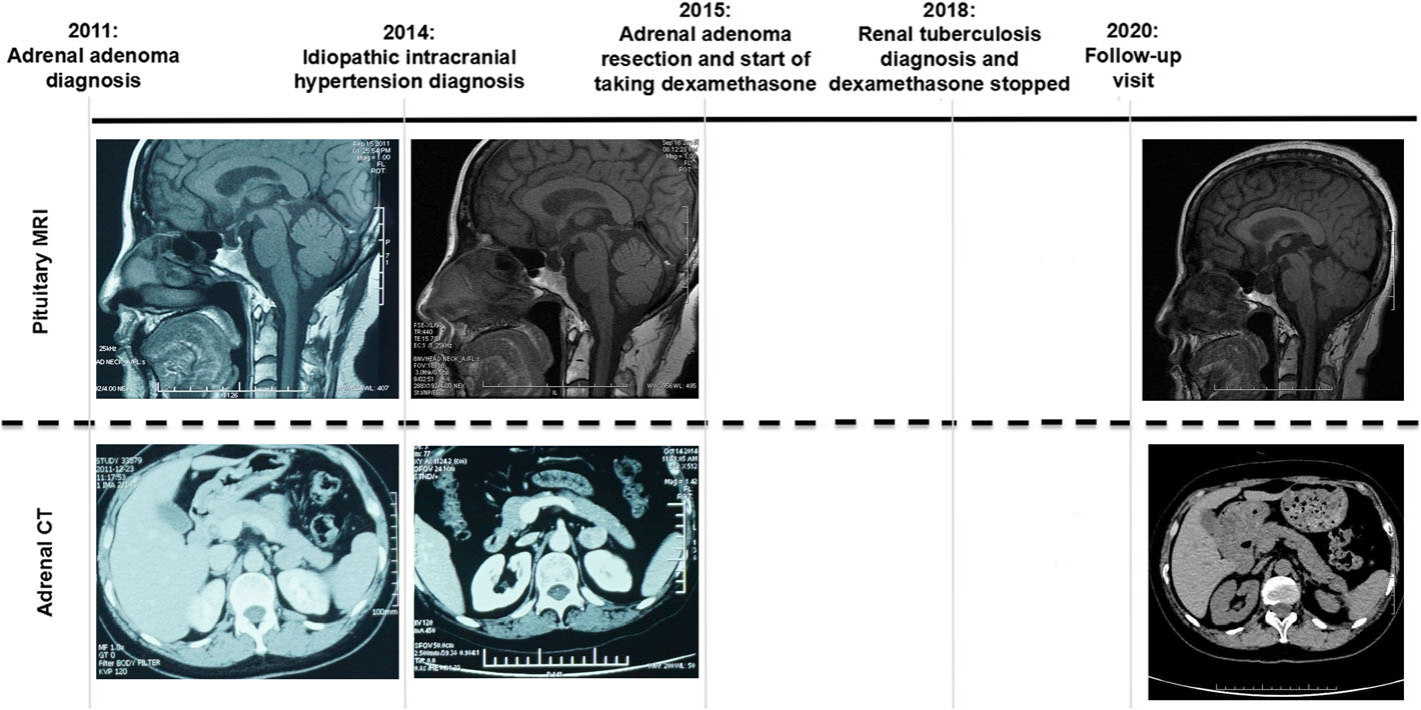
Chronology of diagnosis and treatment. Shown is a summary of diagnosis and treatment. Pituitary MRI shows progressively worsening empty sella and sellar floor erosion from 2011 to 2020. Adrenal CT shows no significant morphological changes in the left adrenal mass from 2011 to 2014. **A** 2011; (**B**) 2014; (**C**) 2020

**Fig. 2:**
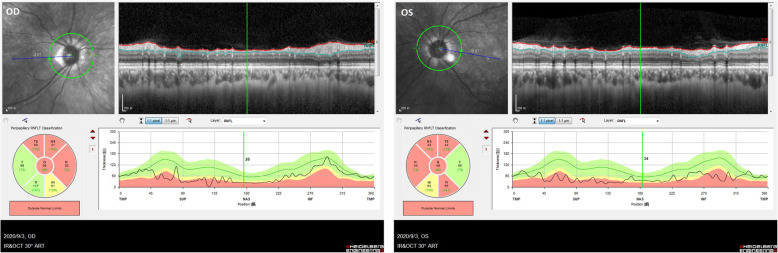
Optical coherence tomography. Optical coherence tomography shows bilateral retinal nerve fiber layer thinning in the peripapillary, more prominent in the left eye

In 2020, a 50-year-old female was admitted to our hospital with persistent blurred vision. In 2011, she presented with high blood pressure. Her blood cortisol (nmol/L) was 324.0 (8 am, reference 171–536), 336.2 (4 pm, reference 64–327) and 303.5 (12 pm). Adrenocorticotrophic hormone (ACTH) was < 0.220 pmol/L (reference 1.10–13.2). She did not receive low-dose dexamethasone suppression test (LDDST) and 24-h urinary cortisol. Pituitary magnetic resonance imaging (MRI) revealed a partial empty sella and adrenal computed tomography (CT) showed a left adrenal mass (Fig. 1A). She was diagnosed with left adrenal adenoma. Her physician suggested making an adrenal CT scan every 3–6 months to observe the change of left adrenal mass. After that, she made frequent medical visits to different hospitals due to various non-specific symptoms such as general fatigue and facial edema. In 2014, she was admitted with truncal obesity, fragile fracture and blurred vision. Her blood cortisol (nmol/L) rose to 429.1, 470.4 and 404.9. ACTH was still < 0.220 pmol/L. LDDST showed that blood cortisol (nmol/L) was 427.8 (8 am, pre-LDDST) and 438.9 (8 am, post-LDDST). Twenty-four-hour urinary free cortisol (nmol/24 h) was 664.86 (reference 100–379). Pituitary MRI showed a complete empty sella and adrenal CT showed no significant changes in the left adrenal mass (Fig. 1B). Bone mineral density by dual energy X-ray absorptiometry established the diagnosis of osteoporosis. Fundus examination confirmed bilateral papilloedema. Lumbar puncture revealed cerebrospinal fluid (CSF) pressure was more than 330 mmH_2_O. She was diagnosed with cortisol-producing adrenal adenoma. Her surgeon considered that the cause of her intracranial hypertension (IH) was unknown and IH was a contraindication for adrenal surgery, so she was referred to multiple superior hospitals for further evaluation. Her cerebral MRI and angiography excluded intracranial mass, aneurysm and venous sinus thrombosis. She was then diagnosed with unexplained IH. In 2015, she underwent adenoma resection. Histological examination and immunohistochemical study of the resected specimen revealed an adrenocortical adenoma on the left side. After the operation, her surgeon told her she needed constant steroid treatment. From 2015 to 2018, she kept taking dexamethasone at least 2 mg daily without her doctors’ consent. Years of seeking medical attention brought a heavy financial burden on her family and the diagnosis of unexplained IH made her lose confidence in treatment. These were why she kept taking dexamethasone and didn’t turn to her doctors again. During this period, she had a further worsening in truncal obesity and blurred vision, and she also developed transient CSF rhinorrhea. In 2018, she was diagnosed with renal tuberculosis because of intermittent fever. Her physician judged glucocorticoid excess was her cause of renal tuberculosis. Her glucocorticoid treatment was then revised to hydrocortisone 20 mg at 8 am and at 4 pm. By 2020, her truncal obesity completely disappeared but blurred vision remained. Pituitary MRI showed a markedly enlarged empty sella and a severe eroded sellar floor, and adrenal CT was normal (Fig. 1C). Fundus examination revealed no papilledema. Optical coherence tomography indicated bilateral optic atrophy (Fig. 2).

## Discussion and conclusions

The patient had been through two stages. Before adrenal adenoma excision, she progressively exhibited a cluster of hallmark symptoms of Cushing’s syndrome from hypertension to truncal obesity and fragile fracture. Laboratory examinations showed that her cortisol circadian rhythm was not present, blood ACTH was undetectable, urinary free cortisol was significantly higher than the normal range, and low doses of dexamethasone didn’t decrease cortisol secretion. Adrenal CT revealed a left adrenal mass. All of these prove that the patient had cortisol-producing adrenal adenoma. In addition, the patient was also found to have IH and worsening empty sella.

There is only one documented case of IH and empty sella from cortisol-producing adrenal adenoma so far [4]. However, this doesn’t mean that IH and empty sella is a rare manifestation of this disorder. As this case shows, the patient’s empty sella had existed when her adrenal adenoma was first found, which suggests that despite the lack of symptoms of IH, her intracranial pressure might have risen above the normal level, because prolonged increased intracranial pressure is one of the many reasons of empty sella. When she was diagnosed with unexplained IH, her empty sella was exacerbated. Meanwhile, she presented with a characteristic cluster of symptoms such as truncal obesity and fragile fracture, and many non-specific symptoms such as general fatigue, facial edema and blurred vision. If her blurred vision was ignored and wasn’t traced to raised intracranial pressure, her IH wouldn’t be found. The disease’s progression implies that cortisol-producing adrenal adenoma can lead to IH and empty sella, and elevated intracranial pressure might be a universal presentation of this disorder but just be neglected due to the lack of noticeable symptoms in early stage or be covered up by other symptoms in later stage. However, this conjecture needs to be further confirmed by more patients with cortisol-producing adrenal adenoma.

After adrenal adenoma excision, the patient continuously misused high-dose dexamethasone, and, as a result, her symptoms of Cushing’s syndrome and IH and her empty sella became more evident and serious. Corticosteroids are frequently used for treating inflammatory and immune-mediated diseases. In some immune-mediated diseases, IH has been described during corticosteroid treatment [5, 6]. Corticosteroids-induced IH is regarded as a rare adverse efect [5]. This patient had no inflammatory or immune-mediated diseases, which can exclude the possibility of IH from a complex interaction between these diseases and corticosteroids. This case suggests that prolonged corticosteroids might inevitably raise intracranial pressure and cause empty sella, but in most cases, these changes are just not to the extent of being perceived. However, this conjecture also needs to be further confirmed by more patients taking corticosteroids.

CSF hypersecretion may have a causative role in glucocorticoid-induced IH. There is a possible mechanism for the effect of glucocorticoids on CSF production [1, 7]. In epithelial choroid plexus cells, glucocorticoids can activate 11β-hydroxysteroid dehydrogenase type 1 (11β-HSD1). 11β-HSD1 converts cortisone to cortisol, and cortisol can activate mineralocorticoid receptors and drive Na^+^/K^+^ ATPase activity, thereby increasing CSF production.

In summary, this case clearly shows how IH and empty sella were gradually formed and accentuated with greater levels of continuous hypercortisolism. The patient seems to be just an extreme case, but it may reveal a general phenomenon: Both cortisol-producing adrenal adenoma and long-term exogenous hypercortisolism could cause varying degrees of IH and empty sella. Clinicians should remain vigilant for this phenomenon. Fundus examination, as an easy to implement, non-invasive and inexpensive screening method for IH, should be routinely used in patients with cortisol-producing adrenal adenoma or exogenous hypercortisolism to identify this complication.

## Data Availability

All data related to this report are kept at Second Hospital of Shanxi Medical University (Shanxi Province, China), and are available from the corresponding author on reasonable request.

## Abbreviations

IH: Intracranial hypertension
IIH: Idiopathic intracranial hypertension
ACTH: Adrenocorticotrophic hormone
LDDST: Low-dose dexamethasone suppression test
MRI: Magnetic resonance imaging
CT: Computed tomography
CSF: Cerebrospinal fluid
11β-HSD1: 11β-hydroxysteroid dehydrogenase type 1
